# Structural equation modeling of parasympathetic and sympathetic response to traffic air pollution in a repeated measures study

**DOI:** 10.1186/1476-069X-12-81

**Published:** 2013-09-23

**Authors:** Emmanuel S Baja, Joel D Schwartz, Brent A Coull, Gregory A Wellenius, Pantel S Vokonas, Helen H Suh

**Affiliations:** 1 Department of Environmental Health, Harvard School of Public Health, Boston, MA, USA; 2 Institute of Clinical Epidemiology, National Institutes of Health, University of the Philippines, Manila, Philippines; 3 Department of Biostatistics, Harvard School of Public Health, Boston, MA, USA; 4 Center for Environmental Health and Technology, Brown University, Providence, RI, USA; 5 VA Normative Aging Study, Veterans Affairs Boston Healthcare System, Boston, MA, USA; 6 Department of Medicine, Boston University, Boston, MA, USA; 7 Environmental Health Program, NORC at the University of Chicago, Boston, MA, USA; 8 Department of Health Sciences, Northeastern University, Boston, MA, USA

**Keywords:** Bayesian, Diabetes, HRV, Obesity, Parasympathetic response, Structural equation models, Sympathetic response and Traffic air pollution

## Abstract

**Background:**

Traffic-related air pollution has been associated to a range of adverse health impacts, including decreased heart rate variability (HRV). The association between traffic-related pollution and HRV, however, has varied by traffic-related or HRV marker as well as by study, suggesting the need for a more comprehensive and integrative approach to examining air pollution-mediated biological impacts on these outcomes. In a Bayesian framework, we examined the effect of traffic pollution on HRV using structural equation models (SEMs) and looked at effect modification by participant characteristics.

**Methods:**

We studied measurements of 5 HRV markers [high frequency (HF), low frequency (LF), 5-min standard deviation of normal-to-normal intervals (SDNN), square root of the mean squared differences of successive normal-to-normal intervals (rMSSD), and LF/HF ratio (LF/HF)] for 700 elderly men from the Normative Aging Study. Using SEMs, we fit a latent variable for traffic pollution that is reflected by levels of carbon monoxide, nitrogen monoxide, nitrogen dioxide, and black carbon (BC) to estimate its effect on latent variable for parasympathetic tone that included HF, SDNN and rMSSD, and the sympathetic tone marker, LF/HF. Exposure periods were assessed using 4-, 24-, 48-, 72-hour moving average pre-visit. We compared our main effect findings using SEMs with those obtained using linear mixed models.

**Results:**

Traffic pollution was not associated with mean parasympathetic tone and LF/HF for all examined moving averages. In Bayesian linear mixed models, however, BC was related to increased LF/HF, an inter quartile range (IQR) increase in BC was associated with a 6.5% (95% posterior interval (PI): -0.7%, 14.2%) increase in mean LF/HF 24-hours later. The strongest association observed was for the 4-hour moving average (10.1%; 95% PI: 3.0%, 17.6%). The effect of traffic on parasympathetic tone was stronger among diabetic as compared to non-diabetic participants. Specifically, an IQR increase in traffic pollution in the 48-hr prior to the clinic visit was associated with a 44.3% (95% PI: -67.7%, -4.2%) lower mean parasympathetic tone among diabetics, and a 7.7% (95% PI: -18.0%, 41.4%) higher mean parasympathetic tone among non-diabetics.

**Conclusions:**

BC was associated with adverse changes LF/HF in the elderly. Traffic pollution may decrease parasympathetic tone among diabetic elderly.

## Background

Studies in air pollution epidemiology have correlated increased levels of traffic-related pollution to increased cardiovascular morbidity and mortality and have also recognized traffic-related pollution as an important risk factor [1]. While the physiological mechanisms of this association have not been fully explained, existing scientific evidence suggests that altered cardiac autonomic control plays a significant role [2,3]. For instance, Schwartz et al. in 2005 found associations between traffic-related pollution exposures and disturbances of autonomic control of the heart as measured through heart rate variability (HRV), a measure of naturally occurring beat-to-beat interval in heart rate [4]. This association between traffic-related pollution and HRV, however, has varied by traffic-related pollutants or HRV markers as well as by study, suggesting the need for a more comprehensive and integrative approach to examining air pollution-mediated biological impacts on these outcomes and in addition to consider multiple traffic-related pollutants and multiple health markers simultaneously.

We used structural equation models (SEMs) to address this issue. SEMs, which represent a family of statistical techniques, allow one to estimate association among multiple latent variables, for example, the relation between traffic pollution and parasympathetic tone. For our analyses, we used latent variables to conceptualize cardiac parasympathetic tone and traffic pollution. These latent variables, traffic and parasympathetic tone, are correlated with measured traffic-related pollutants and markers of HRV, respectively, however they are not observed directly. The latent variables correspond to what is “*common*” among the parameters measured and do not preclude in part different biological processes. Several studies have used SEMs that assessed source-specific health effects of air pollution [5,6] and in health effects of methyl mercury [7] and lead on neurodevelopment [8]. Notably, in models with 3 or more surrogates of the latent exposure, SEMs reduce the attenuation due to measurement error which is an ongoing concern in air pollution studies [9]. This is a typical phenomenon in measurement error corrections, it tends to de-attenuate point estimates coupled with wider confidence intervals due to reflecting additional uncertainty as a result of measurement error correction. The hope is that the total mean squared error (bias squared + variance) is lower than uncorrected estimate.

In this paper, we develop in a Bayesian framework SEMs to examine separately the impact of short-term changes in traffic pollution on parasympathetic tone, and a sympathetic tone marker, the low frequency HRV (LF) to high frequency HRV (HF) ratio (LF/HF), among participants in the Normative Aging Study (NAS). In addition, we look at effect modification by participant characteristics and also compare and contrast the main results of the SEMs to more standard linear mixed models in the Bayesian linear mixed models (BLMMs) framework.

## Methods

### Study population

The NAS is an ongoing longitudinal study of aging launched in 1963 by the Veterans Administration [10]. 2,280 community-dwelling healthy men living in the Greater Boston area were enrolled between 1963 and 1968. Every 3–5 years, after an overnight fast and abstention from smoking, participants visited the clinic for an extensive physical examination, laboratory tests, blood collection, and a self-administered questionnaire on medical history, food intake, medication usage, alcohol consumption, smoking history, and other factors that could affect health.

### Outcome data

The cardiac rhythm was measured for approximately 7 minutes. From November 2000 to December 2009, square root of the mean squared differences of successive normal-to-normal intervals (rMSSD), 5-min standard deviation (SD) of normal-to-normal intervals (SDNN), HF, LF, and LF/HF measurements were obtained during each participant’s regularly scheduled visit [11,12]. To date, HRV measurements have been made for 700 participants, in some cases on multiple occasions (214 participants with one, 216 with two, 259 with three, and 11 with four measurements). A total of 1467 valid measurements were included in our analyses. All participants gave written consent and this study had Institutional Review Board approval.

### Exposure and weather data

We measured concentrations of ambient BC continuously at the Harvard University Countway Library stationary ambient monitoring site that is located < 1 km from the clinical laboratory where subjects were examined. Hourly ambient carbon monoxide (CO), nitrogen monoxide (NO), and nitrogen dioxide (NO_2_) concentrations were obtained from the Massachusetts Department of Environmental Protection (MDEP) for monitoring stations within 20 km of the clinical laboratory, including those in Boston (Bremen, Kenmore Square, North-End, and Roxbury), Waltham and Lynn MA. The pollutant exposure was averaged across all the MDEP monitoring stations; data from the MDEP monitoring stations were used to calculate the mean hourly concentrations of each pollutant. We obtained data on dew point temperature and ambient temperature from the first order National Weather Service station at Boston Logan airport. We calculated apparent temperature (ATemp), a human discomfort index [13], as: ATemp = −2.653 + (0.994 × AT) + (0.0153 × DPT^2^), where DPT is the dew point temperature in Celsius and AT is the ambient temperature in Celsius.

### Statistical analysis

We examined the association between latent traffic pollution and latent parasympathetic tone and between latent traffic pollution and sympathetic tone marker, LF/HF, using SEMs in a Bayesian framework that account for repeated measures. SEMs consist of two key components; a structural model part which shows the association dependencies between two latent constructs, between a latent construct and a measured variable or between two measured variables, and a measurement model part which shows the relation between latent constructs and their indicators/markers [9,14]. HF, SDNN, rMSSD, and LF/HF were log transformed to satisfy the standard SEM assumption that all continuous variables are approximately normally distributed. Descriptive statistics and correlations between HRV markers, traffic-related pollutants and covariates were calculated.

#### Structural equation model with repeated measures in Bayesian framework

We examined associations between traffic-related pollution and markers of parasympathetic tone and sympathetic tone using SEMs, parametric Directed Acyclic Graphs (DAGs) that contain specified paths connecting observed and latent variables [9,14]. SEMs in Bayesian framework consist of (1) distributional assumptions on the latent variables, (2) prior distributions for the parameters, and (3) probability models for the observed variables given latent variables and model parameters. SEMs also specify linear models relating the unknown means of the latent traffic pollution, sympathetic tone marker LF/HF and latent parasympathetic tone variables to fixed covariates (e.g. confounding factors), other latent variables, and a subject level random intercept to accommodate the repeated measures design of the study. For the effect of traffic pollution on both LF/HF and the latent parasympathetic tone, exposure periods were assessed using 4-hr, 24-hr, 48-hr and 72-hr moving average pre-visit, given results from previous studies that show effects at similar exposure windows [4,11].

**Figure 1:**
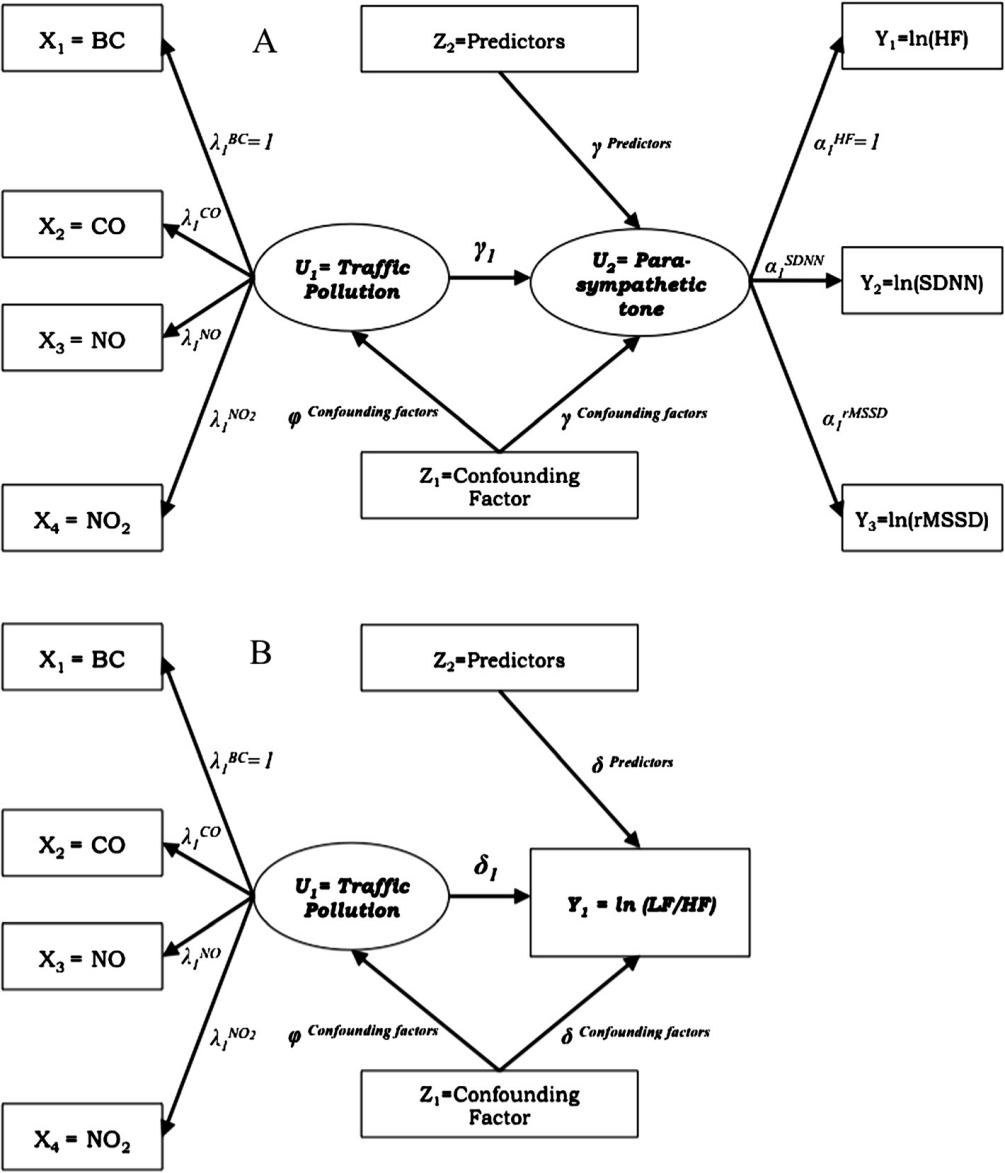
**Path diagrams of the structural equation models. (A)** The effect of traffic pollution on parasympathetic tone. **(B)** The effect of traffic pollution on sympathetic tone marker, LF/HF. Ellipses are used to denote latent constructs, rectangles are used to denote the observed variables measuring and affecting these constructs, and single-headed arrows are used to denote directional relationships, from predictor to outcome.

For the measurement part of the SEM, we specified measurement models describing the relationships between observed measures and latent variables. The relationship between the traffic-related observed measures (BC, CO, NO, and NO_2_) and the latent exposure variable *Traffic* was first modeled (Figure 1).

For moving average concentration pre-visit measurement of participants *i* = 1, …*I*, of traffic-related pollutants *j* = 1, …*J*, on participant’s scheduled time visits *t* = 1, …*T*,

(1) $$X_{\mathrm{ijt}}\sim N\;\left(\mu_{\mathrm{ijt}}{}^{X},\tau_{j}{}^{X}\right)$$

where *X*_*ijt*_ is the normally distributed traffic-related exposure variable for moving average concentration pre-visit measurement of participant *i* of traffic-related pollutant *j* on participant’s scheduled time visit *t*, with unknown mean *μ*_*ijt*_^*X*^ and precision (1/variance) *τ*_*j*_^*X*^. The mean exposure of traffic-related pollutant *j*, *μ*_*ijt*_^*X*^, was related to the latent exposure variable *Traffic*_*it*_ through a linear model,

(2) $$\mu_{\mathrm{ijt}}{}^{X}=\lambda_{0j}+\lambda_{1j}\mathrm{Traffi}c_{\mathrm{it}}$$

where *Traffic*_*it*_ is the normally distributed latent exposure variable *Traffic* for participant *i* for scheduled time visit *t*, with unknown mean *μ*_*it*_^*Traffic*^ and precision (1/variance) *τ*^*Traffic*^,

(3) $$\mathrm{Traffi}c_{\mathrm{it}}\sim N\left(\mu_{\mathrm{it}}{}^{\mathrm{Traffic}},\tau^{\mathrm{Traffic}}\right).$$

The mean *Traffic* exposure, *μ*_*it*_^*Traffic*^, was related to confounding factors, through a linear model,

(4) $$\begin{array}{l} \mu_{\mathrm{it}}^{\mathrm{Traffic}}=\varphi_{1}\mathrm{Sine}\;\left[2\pi \theta \left(t\right)/365.24\right]+\varphi_{2}\mathrm{Cosine}\;[2\pi \theta \left(t\right)/365.24]\\ \;+\varphi_{3}\mathrm{Residual}{\left(\mathrm{ATemp}\right)}_{t}+\varphi_{4}\mathrm{Residua}l^{2}{\left(\mathrm{ATemp}\right)}_{t} \end{array}$$

where *θ* (t) is the calendar day of the year the participant visited the clinic, the sine and cosine terms capture seasonal trends in traffic pollution, ATemp is apparent temperature averaged across the same exposure window, and residual (ATemp) is the residual of a model regressing ATemp against sine and cosine terms of the calendar day of the year in the study. This approach was taken because temperature data was highly seasonal, and this avoids substantial collinearity between the seasonal terms and temperature, which would otherwise be present in the model. Furthermore, Eq. (4) looks like it doesn’t vary with participant *i* but it does vary because the scheduled time visits *t* are unique to that participant.

The relationship between the latent outcome variable, and its observed markers was also modeled. The HRV markers HF, SDNN and rMSSD were used as the markers for the latent *Parasympathetic tone* variable. For HRV measurement of participants *i* = 1, …*I*, of HRV parasympathetic tone markers *k* = 1, …*K*, on participant’s scheduled time visits *t* = 1, …*T*,

(5) $$Y_{\mathrm{ikt}}\sim N\left(\mu_{\mathrm{ikt}}^{Y},\tau_{k}^{Y}\right)$$

where *Y*_*ikt*_ represents the normally distributed markers of parasympathetic tone for HRV measurement of participant *i* of parasympathetic tone marker *k* on participant’s scheduled time visit *t*, with unknown mean *μ*_*ikt*_^*Y*^ and precision (1/variance) *τ*_*k*_^*Y*^. For parasympathetic tone marker *k*, the mean outcome, *μ*_*ikt*_^*Y*^, was related to the latent outcome variable *Parasympathetic tone* through a linear model,

(6) $$\mu_{\mathrm{ikt}}^{Y}=\alpha_{0k}+\alpha_{1k}\mathrm{Parasympathetic}\;\mathrm{ton}e_{\mathrm{it}}$$

where *Parasympathetic tone*_*it*_ is the normally distributed latent outcome variable *Parasympathetic tone* for scheduled time visit *t* for participant *i*, with unknown mean *μ*_*it*_^*Parasympathetic tone*^ and precision (1/variance) *τ*^*Parasympathetic tone*^,

(7) $$\begin{array}{l} \mathrm{Parasympathetic}\;\mathrm{ton}e_{\mathrm{it}}\sim N\\ \;\left(\mu_{\mathrm{it}}^{\mathrm{Parasympathetic}\;\mathrm{tone}},\tau^{\mathrm{Parasympathetic}\;\mathrm{tone}}\right). \end{array}$$

Furthermore, for LF/HF measurement of participants *i* = 1, …*I*, on participant’s scheduled time visits *t* = 1, …*T*,

(8) $$\mathrm{LF}/{\mathrm{HF}}_{\mathrm{it}}\sim N\left(\mu_{\mathrm{it}}^{\mathrm{LF}/\mathrm{HF}},\tau^{\mathrm{LF}/\mathrm{HF}}\right)$$

where LF/HF_*it*_ is the normally distributed sympathetic tone marker outcome for participant *i* for scheduled time visit *t*, with unknown mean *μ*_*it*_^LF/HF^ and precision (1/variance) *τ*^LF/HF^.

The mean *Parasympathetic tone* outcome, *μ*_*it*_^*Parasympathetic tone*^ was related to the latent exposure variable *Traffic*_*it*_, confounding factors, predictors of the outcome variable, and a subject level random intercept, *a*_*i*_, through a linear model,

(9) $$\begin{array}{l} \mu_{\mathrm{it}}^{\mathrm{Parasympathetic}\;\mathrm{tone}}=\gamma_{1}\mathrm{Traffi}c_{\mathrm{it}}+\gamma_{2}X_{1\mathrm{it}}+\ldots +\gamma_{m+1\;}X_{\mathrm{mit}}\\ \;+\gamma_{m+2\;}\mathrm{Sine}\left[2\pi \theta \left(t\right)/365.24\right]\;+\gamma_{m+3\;}\mathrm{Cosine}\left[2\pi \theta \left(t\right)/365.24\right]\\ \;+\gamma_{m+4\;}\mathrm{Residual}{\left(\mathrm{ATemp}\right)}_{t}+\gamma_{m+5\;}\mathrm{Residua}l^{2}{\left(\mathrm{ATemp}\right)}_{t}+a_{i}. \end{array}$$

In addition, the mean LF/HF outcome, *μ*_*it*_^LF/HF^, was also related to the latent exposure variable *Traffic*_*it*_, predictors of the outcome variable, confounding factors, and a subject level random intercept, *b*_*i*_, through a linear model,

(10) $$\begin{array}{l} \mu_{\mathrm{it}}^{\mathrm{LF}/\mathrm{HF}}=\delta_{1}\mathrm{Traffi}c_{\mathrm{it}}+\delta_{2}X_{1\mathrm{it}}+\ldots +\delta_{m+1\;}X_{\mathrm{mit}}\\ \;+\delta_{m+2\;}\mathrm{Sine}\left[2\pi \theta \left(t\right)/365.24\right]\;+\delta_{m+3\;}\mathrm{Cosine}\left[2\pi \theta \left(t\right)/365.24\right]\\ \;+\delta_{m+4\;}\mathrm{Residual}{\left(\mathrm{ATemp}\right)}_{t}+\delta_{m+5\;}\mathrm{Residua}l^{2}{\left(\mathrm{ATemp}\right)}_{t}+b_{i}. \end{array}$$

For Eqs. (9) and (10), the fixed covariates (*X*_*1*_*, …, X*_*m*_) included age, fasting blood glucose, body mass index (BMI), smoking status (ever/never), room temperature, sitting mean arterial blood pressure, ≥ 2 servings of alcohol per day (yes/no), and cardiac medication usage (beta blocker, angiotensin converting enzyme inhibitor, and calcium channel blocker). These covariates were chosen a priori as potentially important predictors of both *Parasympathetic tone* and LF/HF. To account for seasonal variation in *Parasympathetic tone* and LF/HF, a function of calendar date {sine [2πθ(t)/365.24] + cosine [2πθ(t)/365.24], where *θ* (t) is the calendar day of the year the participant visited the clinic} was used. To account for temperature variation in both *Parasympathetic tone* and LF/HF, we regressed ATemp on sine [2πθ(t)/365.24] + cosine [2πθ(t)/365.24] and used the linear [Residual (ATemp)_t_] and quadratic [Residual^2^ (ATemp)_t_] residuals of the equation as part of the fixed effects of the model. We also included a subject level random intercept, *a*_*i*_ for Eq. (9) and *b*_*i*_ for Eq. (10), to represent subject-specific permanent effects for each participant to accommodate the repeated measures design of the study. Moreover, Eqs. (9) and (10) implicitly depend on participant *i* because the scheduled time visits *t* depend on participant *i.* To evaluate obesity as an effect modifier, subjects were classified into two groups according to BMI (obese: BMI ≥ 30 vs. non-obese: BMI < 30). We also assessed effect modification by a history of diabetes (diabetic vs. non-diabetic) and by the four possible combinations of obesity and history of diabetes (diabetic and obese, diabetic and non-obese, non-diabetic and obese, and non-diabetic and non-obese). We included latent interaction term constructs between the dichotomized modifier variable and traffic pollution in Equations (9) and (10).

For model identifiability, the location of all the latent variables was centered and set to 0 (i.e. *δ*_*0*_*, φ*_*0*_ and *γ*_*0*_ = 0). A scale must be specified for each latent variable because latent variables were constructed from multiple measured parameters, which have different scales of variation. We chose HF as the reference scale for *Parasympathetic tone* and BC as the reference scale for *Traffic* pollution. In order to set the reference scale for *Parasympathetic tone* and *Traffic* and for identifiability and interpretation of these latent variables, α_1_^HF^ and λ_1_^BC^, were also constrained to be equal to 1 [6]. For the DAG diagram of the SEMs, see Figure 1.

#### Linear mixed models in Bayesian framework

In order to compare the results from the SEM in Bayesian framework with repeated measures to those obtained from a standard univariate analysis, associations between traffic-related air pollutant BC and mean markers of parasympathetic tone (HF) and sympathetic tone (LF/HF) were estimated using linear mixed models with random subject-specific intercepts in Bayesian linear mixed models (BLMMs) framework. Additionally, we compared the results from the above BLMMs to Frequentist framework estimates (Frequentist linear mixed models, LMMs) obtained from LME in R (http://www.r-project.org). The same fixed and random covariates used in SEMs were included in all the LMMs and BLMMs. To allow more direct comparison for the SEMs, BLMMs, and LMMs, effect size estimates were reported for an inter-quartile range (IQR) increase in BC (reference pollutant of latent variable *Traffic* pollution).

We specified a model for the measured health effect outcomes, HF and LF/HF. The means of both HF and LF/HF for participant *i* on participant’s scheduled time visit *t*, *E[HF*_*it*_*]* and *E[LF/HF*_*it*_*]*, respectively, was related to BC, confounding factors and predictors of the health effect outcomes, and a subject level random intercept, *c*_*i*_ or *d*_*i*_, through a linear model,

(11) $$\begin{array}{l} E\left[HF_{\mathrm{it}}\right]=\beta_{0}+\beta_{1}BC_{\mathrm{it}}+\beta_{2}X_{1\mathrm{it}}+\ldots +\beta_{m+1\;}X_{\mathrm{mit}}\\ \;+\beta_{m+2}\mathrm{Sine}\left[2\pi \theta \left(t\right)/365.24\right]\;+\beta_{m+3}\mathrm{Cosine}\left[2\pi \theta \left(t\right)/365.24\right]\\ \;+\beta_{m+4}\mathrm{Residual}{\left(\mathrm{ATemp}\right)}_{t}+\beta_{m+5}\mathrm{Residua}l^{2}{\left(\mathrm{ATemp}\right)}_{t}+c_{i} \end{array}$$

(12) $$\begin{array}{l} E\left[\mathrm{LF}/HF_{\mathrm{it}}\right]=\rho_{0}+\rho_{1}BC_{\mathrm{it}}+\rho_{2}X_{1\mathrm{it}}+\ldots +\rho_{m+1\;}X_{\mathrm{mit}}\\ \;+\rho_{m+2}\mathrm{Sine}\left[2\pi \theta \left(t\right)/365.24\right]\;+\rho_{m+3}\mathrm{Cosine}\left[2\pi \theta \left(t\right)/365.24\right]\\ \;+\rho_{m+4}\mathrm{Residual}{\left(\mathrm{ATemp}\right)}_{t}+\rho_{m+5}\mathrm{Residua}l^{2}{\left(\mathrm{ATemp}\right)}_{t}+d_{i}. \end{array}$$

In terms of Bayesian inference, results were quantified and characterized in terms of the strength of the following hypothesized relationships: (1) inverse relationships between BC and HRV marker HF, and between *Traffic* pollution and *Parasympathetic tone*, and (2) positive relationships between BC and the HRV marker LF/HF, and between *Traffic* pollution and sympathetic tone marker, LF/HF. In addition, results were presented as probability statements, providing evidence that coefficients describing *Traffic* pollution (*γ*_*1*_ and *δ*_*1*_) or BC (*β*_*1*_ and *ρ*_*1*_) are either negative or positive depending on the hypothesized relationship. See Additional file 1 for details for implementation particulars and description of prior distributions of parameters, and see Additional file 2 for the WinBUGS code.

## Results

700 NAS eligible study participants with valid HRV measurements available were included for analysis. Subjects were male with a mean age of 75.0 years (SD = 6.7 years), were mostly ever cigarette smokers (69.5%), and were mostly overweight with a mean BMI of 28.1 kg/m^2^ (SD = 4.2 kg/m^2^). Table 1 shows the descriptive statistics for ambient air pollutants and HRV measures. Traffic pollutant concentrations, averaged over 24 hours before blood collection, were strongly correlated with CO and NO_2_ having the highest correlation (Spearman correlation coefficient, ρ = 0.63). The correlation between HRV markers was highest for HF and rMSSD (ρ = 0.93). The LF/HF marker is negatively correlated with the rest of the HRV markers.

**Table 1:** Summary statistics for heart rate variability (HRV) and 24-hr moving average ambient air pollutant measures (November 2000 to December 2009)

	**Average**	**Median**	**SD**	**5%**	**95%**	**IQR**
Air pollution measure						
BC (μg/m^3^)	0.83	0.73	0.43	0.31	1.62	0.55
CO (ppm)	0.43	0.40	0.26	0.11	0.92	0.31
NO (ppm)	0.02	0.01	0.02	0.01	0.06	0.01
NO_2_ (ppm)	0.02	0.02	0.01	0.01	0.03	0.01
HRV measure^a^						
ln(SDNN)	3.7	3.6	0.8	2.6	5.2	1.0
ln(r-MSSD)	3.5	3.2	1.1	2.1	5.6	1.5
ln(HF)	4.9	4.6	2.0	2.2	8.8	2.7
ln(LF)	4.8	4.6	1.6	2.6	8.0	2.0
ln(LF/HF)	−0.1	0.0	1.1	−2.0	1.7	1.5

^a^log transformed HRV measures; SDNN, 5-min SD of normal-to-normal intervals; r-MSSD, square root of the mean squared differences of successive normal-to-normal intervals; HF, high-fequency HRV; LF, low-frequency HRV; LF/HF, ratio of LF and HF.

### Traffic pollution and parasympathetic tone measurement models

**Figure 2:**
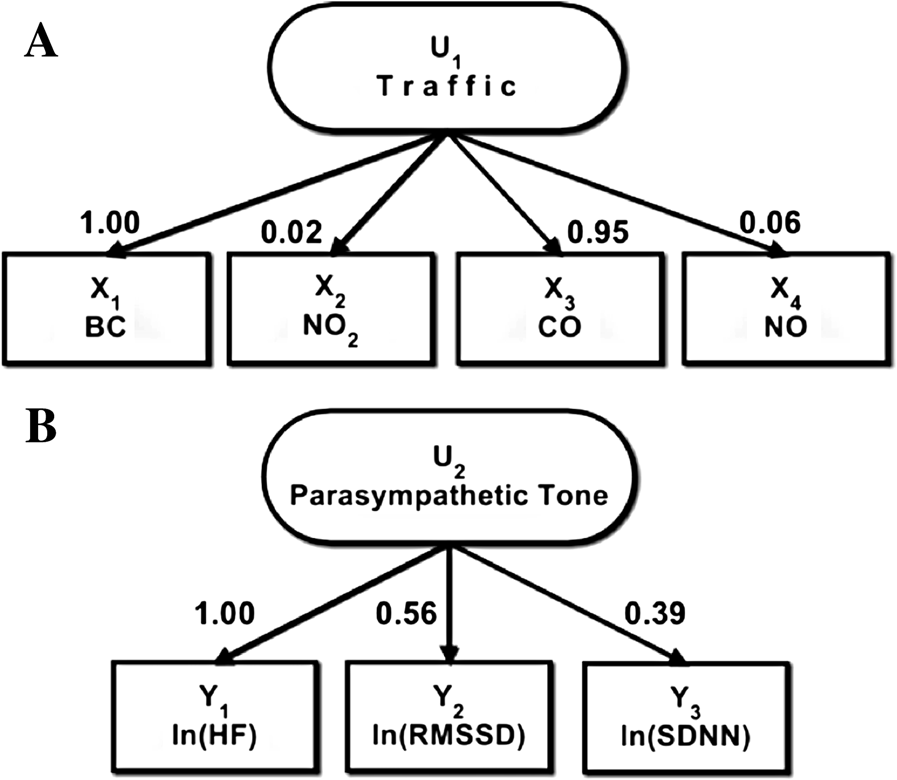
**Measurement models of traffic and parasympathetic tone.** Relation of latent variables of **(A)***Traffic* and **(B)***Parasympathetic tone* to marker variables as factor loadings values. 24-hr moving average ambient air pollution measurements from November 2000 to December 2009.

Table 2 tabulates the factor loadings (*λ*_*1j*_ coefficients of Equation 2) of the *Traffic Pollution* measurement model and the corresponding variance of the *Traffic* latent (1/*τ*^*Traffic*^ of Equation 3) and its marker variables (1/*τ*_*j*_^*X*^ of Equation 1). Moreover, for the measurement model for the relation of the *Parasympathetic tone* latent variable to its various marker variables, the factor loadings (*α*_*1k*_ coefficients of Equation 6) and the variance of the *Parasympathetic tone* (1/*τ*^*Parasympathetic tone*^ of Equation 7) and its marker variables (1/*τ*_*k*_^*Y*^ of Equation 5) were also summarized in Table 2. Across all the different moving averages, the results of the factor loading estimates (*α*_*1k*_) for the relation of *Parasympathetic Tone* to its markers were very similar. In using BC as the reference marker, for the 4-hr moving average exposure, the highest reliability measure of *Traffic* was CO [coefficient as factor loading of 0.65; 95% Posterior Interval (PI): 0.59 to 0.72], while the lowest reliability measure of *Traffic* was NO_2_ (0.01; 95% PI: 0.01 to 0.01). Furthermore, for the 24-hr moving average exposure, CO predominantly represented *Traffic* (0.95; 95% PI: 0.86 to 1.05) while NO_2_ was still the lowest reliability measure of *Traffic* (0.02; 95% PI: 0.02 to 0.02) (Figure 2A). In addition, in using HF as the reference marker for the 24-hr moving average exposure, *Parasympathetic tone* was strongly represented by rMSSD with a coefficient as factor loading of 0.56 (95% PI: 0.55 to 0.57) (Figure 2B). In addition, for the 4-hr moving average exposure, the variance of latent *Parasympathetic Tone* variable was higher (1.720; 95% PI: 1.535 to 1.921) compared to the variance of the latent *Traffic* variable (0.137; 95% PI: 0.112 to 0.165). For the HRV markers, for a 4-hr moving average exposure, the variance of HF was the highest (0.309; 95% PI: 0.273 to 0.346) and the variance of rMSSD was the lowest of the 3 markers (0.052; 95% PI: 0.043 to 0.062). Of the 4 traffic-related pollutants, the variance of the 4-hr moving average BC was the highest (0.394; 95% PI: 0.364 to 0.425), while NO_2_ was the lowest in variance (4.3E-5; 95% PI: 4.0E-5 to 4.7E-5) for all the pollutants.

**Table 2:** **Factor loadings (*λ***_***1j ***_**and *α***_***1k ***_**coefficients) of the measurement models for the relation of *Traffic *and *Parasympathetic Tone *latent variables to its various marker variables**^**a **^**and the corresponding variance of latent (1/*τ***^***Traffic ***^**and 1/*τ***^***Parasympathetic Tone***^**) and its marker (1/*τ***_***j***_^***X ***^**and 1/*τ***_***k***_^***Y***^**) variables**

**Moving average**	**Latent/ Markers**	**Factor loading (*λ***_***1j***_**) (95% PI)**^**b**^	**Variance (95% PI)**	**Latent/Markers**	**Factor loading (*α***_***1k***_**) (95% PI)**^**b**^	**Variance (95% PI)**
04-hour	Traffic/		0.137 (0.112, 0.165)	Parasympathetic		1.720 (1.535, 1.921)
	BC^c^	1.00	0.394 (0.364, 0.425)	Tone/		
	CO	0.65 (0.59, 0.72)	0.010 (0.006, 0.013)	HF^c^	1.00	0.309 (0.273, 0.346)
	NO	0.05 (0.05, 0.06)	3.0E-4 (2.7E-4, 3.4E-4)	SDNN	0.39 (0.38, 0.40)	0.073 (0.066, 0.080)
	NO_2_	0.01 (0.01, 0.01)	4.3E-5 (4.0E-5, 4.7E-5)	rMSSD	0.56 (0.55, 0.57)	0.052 (0.043, 0.062)
24-hour	Traffic/		0.036 (0.029, 0.044)	Parasympathetic		1.728 (1.542, 1.930)
	BC	1.00	0.127 (0.118, 0.136)	Tone/		
	CO	0.95 (0.86, 1.05)	0.010 (0.008, 0.011)	HF	1.00	0.305 (0.271, 0.342)
	NO	0.06 (0.06, 0.07)	1.1E-4 (1.0E-4, 1.2E-4)	SDNN	0.39 (0.38, 0.40)	0.074 (0.067, 0.081)
	NO_2_	0.02 (0.02, 0.02)	3.2E-5 (3.0E-5, 3.4E-5)	rMSSD	0.56 (0.55, 0.57)	0.051 (0.042, 0.060)
48-hour	Traffic/		0.023 (0.019, 0.029)	Parasympathetic		1.754 (1.568, 1.951)
	BC	1.00	0.094 (0.087, 0.102)	Tone/		
	CO	1.01 (0.91, 1.12)	0.009 (0.007, 0.010)	HF	1.00	0.306 (0.270, 0.343)
	NO	0.06 (0.06, 0.07)	7.7E-5 (7.0E-5, 8.5E-5)	SDNN	0.39 (0.38, 0.40)	0.073 (0.067, 0.080)
	NO_2_	0.02 (0.02, 0.03)	2.8E-5 (2.6E-5, 3.0E-5)	rMSSD	0.56 (0.55, 0.57)	0.052 (0.042, 0.061)
72-hour	Traffic/		0.016 (0.013, 0.020)	Parasympathetic		1.753 (1.569, 1.953)
	BC	1.00	0.069 (0.064, 0.075)	Tone/		
	CO	1.11 (1.00, 1.24)	0.008 (0.006, 0.009)	HF	1.00	0.304 (0.268, 0.340)
	NO	0.06 (0.06, 0.07)	5.9E-5 (5.4E-5, 6.5E-5)	SDNN	0.39 (0.38, 0.40)	0.074 (0.067, 0.081)
	NO_2_	0.03 (0.02, 0.03)	2.5E-5 (2.4E-5, 2.7E-5)	rMSSD	0.56 (0.55, 0.57)	0.051 (0.042, 0.061)

^a^HF, high frequency; SDNN, 5-min standard deviation of normal-to-normal intervals; rMSSD, square root of the mean squared differences of successive normal-to-normal intervals; BC, black carbon; CO, carbon monoxide; NO, nitrogen monoxide; NO_2_, nitrogen dioxide.

^b^95% PI: 95% Posterior Interval.

^c^Reference marker.

### Effect of traffic pollution on parasympathetic tone and sympathetic tone marker LF/HF

Table 3 shows the estimated percent change in parasympathetic tone per IQR increase in BC, the reference pollutant of the latent traffic pollution, for various hourly-moving averages. For the 48-hr pre-visit moving average, a change of −3.0% (95% PI: -24.7% to 25.4%) in parasympathetic tone (reference marker: HF) was associated with an IQR increase in BC (reference pollutant of traffic pollution exposure). Results showed that the 4-hr posterior distribution gave the smallest variance compared to other hourly moving averages. Table 3 also presents the estimated percent change in LF/HF, a marker of sympathetic tone, per IQR increase in BC (reference pollutant of latent traffic pollution) for various hourly-moving averages. We found a change of 6.4% (95%PI: -9.5% to 24.3%) in LF/HF associated with an IQR increase in BC (reference pollutant of traffic pollution) 48-hr pre-visit. The distributions for SEM also showed that the 4-hr posterior distribution gave the smallest variance compared to other hourly moving averages and as the averaging period increases from 4-hr to 72-hr, the variability or spread of the distribution also increased, as evidenced by the wider posterior probability distributions. Furthermore, in both the SEM and BLMM approaches, the posterior probabilities characterized and quantified strong positive relationships in all the exposure windows (4-hr, 24-hr, 48-hr, and 72-hr) between *Traffic* pollution and sympathetic tone marker, LF/HF, and between BC and HRV marker LF/HF (posterior probabilities ≥ 0.78).

**Table 3:** **Posterior estimates of % change in health effect outcome associated with an IQR**^**a **^**increase in traffic (left) or BC (right)**

**S T R U C T U R A L E Q U A T I O N M O D E L (SEM)**					**B A Y E S I A N L I N E A R M I X E D M O D E L (BLMM)**				
**Exposure/ Outcome**	**Moving average**	**% change in mean**	**95% PI**	**Posterior probability**	**Exposure/ Outcome**	**Moving average**	**% change in mean**	**95% PI**	**Posterior probability**
Traffic/	4-hr	0.1	(−19.0, 25.0)	0.50^c^	BC/	4-hr	−4.8	(−14.8, 6.3)	0.81^e^
Para-	24-hr	3.2	(−19.4, 32.2)	0.40^c^	HF^d^	24-hr	−6.9	(−16.9, 4.5)	0.89^e^
sympathetic	48-hr	−3.0	(−24.7, 25.4)	0.59^c^		48-hr	−3.7	(−13.9, 7.9)	0.74^e^
tone^b^	72-hr	2.8	(−19.2, 29.1)	0.40^c^		72-hr	−2.9	(−13.5, 8.9)	0.70^e^
Traffic/	4-hr	6.6	(−6.7, 21.8)	0.83^g^	BC/	4-hr	10.1	(3.0, 17.6)	0.99^i^
LF/HF^f^	24-hr	7.2	(−8.0, 25.1)	0.82^g^	LF/HF^h^	24-hr	6.5	(−0.7, 14.2)	0.96^i^
	48-hr	6.4	(−9.5, 24.3)	0.78^g^		48-hr	4.7	(−2.3, 12.2)	0.89^i^
	72-hr	7.1	(−9.3, 26.0)	0.80^g^		72-hr	2.7	(−4.2, 10.1)	0.78^i^

^a^Interquartile range (IQR): 4-hr = 0.88; 24-hr = 0.52; 48-hr = 0.43; 72-hr = 0.36.

^b^SEM: effect of traffic pollution on parasympathetic tone.

^c^Posterior probability that *γ*_*1*_ < 0.

^d^BLMM: effect of black carbon on HF.

^e^Posterior probability that *β*_*1*_ < 0.

^f^SEM: effect of traffic pollution on LF/HF.

^g^Posterior probability that *δ*_*1*_ > 0.

^h^BLMM: effect of black carbon on LF/HF.

^i^Posterior probability that *ρ*_*1*_ > 0.

### Effect modification

**Figure 3:**
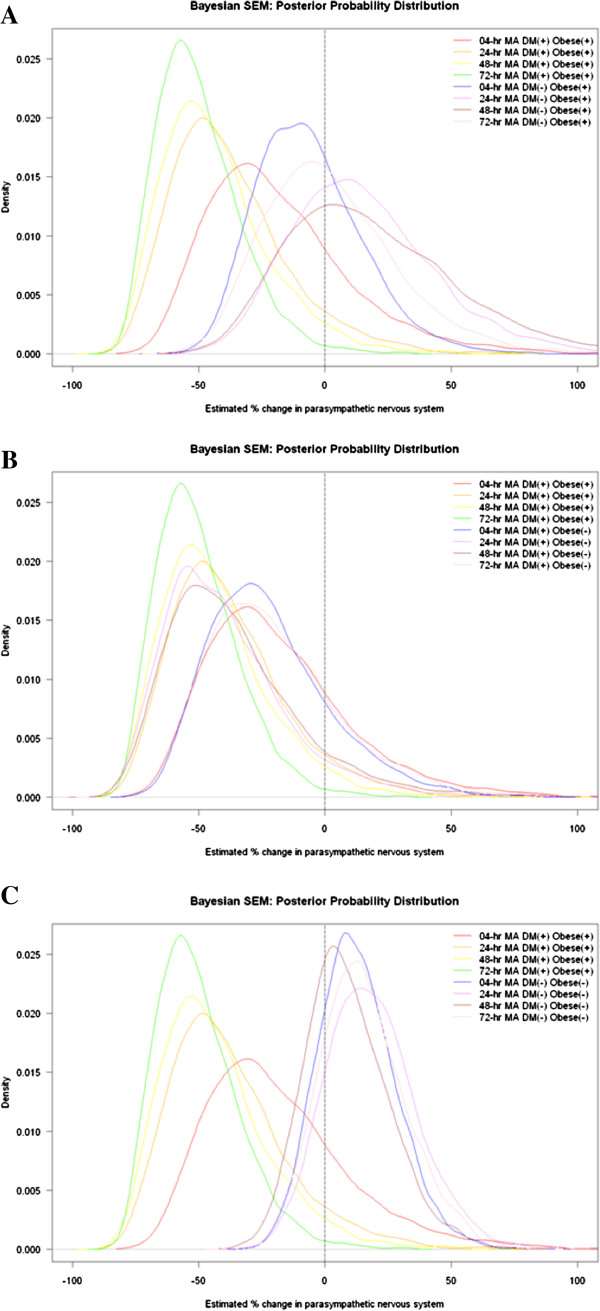
**Adjusted density plots of posterior estimated percentage change in parasympathetic tone associated with an inter quartile range (IQR) increase in traffic pollution at different hourly moving averages (MA), by patient characteristics [diabetic status (doctor’s diagnosis of diabetes or fasting blood glucose (FBG) >126 mg/dL, vs. no diagnosis or FBG ≤126 mg/dL), and obesity (body mass index (BMI) ≥ 30, <30)]. (A)** Diabetic vs. non-diabetic among obese participants, **(B)** obese vs. non-obese among diabetic participants, and **(C)** diabetic and obese participants vs. non-diabetics and non-obese participants.

We assessed whether being diabetic, obese, or being both obese and diabetic, modified the effect of traffic pollution on parasympathetic tone and sympathetic tone marker, LF/HF. An IQR increase in BC (reference pollutant of latent traffic pollution) 48-hr pre-visit was associated with a −44.3% (95% PI: -67.7% to −4.2%) change in mean parasympathetic tone (reference marker: HF) among participants with diabetes, while for the non-diabetic participants the association was a 7.7% change in mean parasympathetic tone (95% PI: -18.0% to 41.4%). The effect of traffic pollution on parasympathetic tone was also stronger for diabetic versus non-diabetic and obese versus non-obese participants (for details, see Table 4). Furthermore, among obese participants, the association of parasympathetic tone with traffic pollution was stronger for diabetic participants versus non-diabetic participants at all exposure periods (Figure 3A). However, among diabetic participants, the association was similar for obese participants versus non-obese participants across all examined exposure windows, except for the 72-hr moving average where the effect was two times stronger for obese versus non-obese participants (Figure 3B). In addition, Figure 3C compares the effect of traffic on parasympathetic tone among participants that are both diabetic and obese versus participants that are both non-diabetics and non-obese and the effects were stronger for participants that are both diabetic and obese (for the effect of traffic on sympathetic tone marker (LF/HF) effect modification results, see Additional file 3 for details).

**Table 4:** **Adjusted posterior estimates of % change in mean parasympathetic tone associated with an IQR**^**a **^**increase in traffic, by patient characteristics [diabetic status (doctor’s diagnosis of diabetes or fasting blood glucose (FBG) >126 mg/dL, vs. no diagnosis or FBG ≤126 mg/dL), and obesity (body mass index (BMI) ≥ 30, <30)]**

**S T R U C T U R A L E Q U A T I O N M O D E L**									
**Effect modifier**	**Moving average**	**% change in mean**	**95% PI**	**Posterior probability**^**b**^	**Effect modifier**	**Moving average**	**% change in mean**	**95% PI**	**Posterior probability**^**b**^
Diabetic	4-hr	−22.1	(−50.9, 22.7)	0.86	Diabetic	4-hr	−23.2	(−60.0, 51.1)	0.78
	24-hr	−41.9	(−66.3, -0.4)	0.98	Obese	24-hr	−42.1	(−72.5, 18.5)	0.93
	48-hr	−44.3	(−67.7, -4.2)	0.98		48-hr	−47.0	(−74.7, 7.5)	0.96
	72-hr	−42.2	(−65.2, -6.7)	0.99		72-hr	−52.7	(−74.8, -11.6)	0.99
Non-Diabetic	4-hr	5.9	(−15.7, 34.0)	0.31	Non-Diabetic	4-hr	−8.6	(−40.0, 40.2)	0.66
	24-hr	14.8	(−11.7, 49.1)	0.15	Obese	24-hr	11.7	(−31.7, 81.7)	0.32
	48-hr	7.7	(−18.0, 41.4)	0.30		48-hr	4.6	(−33.2, 100.2)	0.32
	72-hr	10.2	(−15.4, 43.4)	0.23		72-hr	−0.8	(−39.3, 63.4)	0.51
Obese	4-hr	−13.3	(−40.5, 26.2)	0.78	Diabetic	4-hr	−25.6	(−59.1, 35.4)	0.83
	24-hr	−7.8	(−38.8, 39.3)	0.65	Non-Obese	24-hr	−44.2	(−74.3, 21.3)	0.93
	48-hr	−13.2	(−45.5, 36.9)	0.72		48-hr	−41.3	(−73.7, 30.9)	0.91
	72-hr	−26.8	(−52.5, 12.1)	0.92		72-hr	−24.6	(−60.9, 43.2)	0.81
Non-Obese	4-hr	6.3	(−16.7, 36.1)	0.32	Non-Diabetic	4-hr	11.8	(−14.3, 45.7)	0.20
	24-hr	7.6	(−19.0, 43.5)	0.31	Non-Obese	24-hr	17.1	(−13.4, 57.0)	0.15
	48-hr	1.0	(−24.0, 35.4)	0.48		48-hr	7.7	(−19.6, 46.5)	0.31
	72-hr	8.5	(−17.7, 44.2)	0.28		72-hr	13.9	(−14.9, 52.3)	0.19

^a^Interquartile range (IQR): 4-hr = 0.88; 24-hr = 0.52; 48-hr = 0.43; 72-hr = 0.36.

^b^Posterior probability that *γ*_*1*_ < 0.

### Model comparison

**Figure 4:**
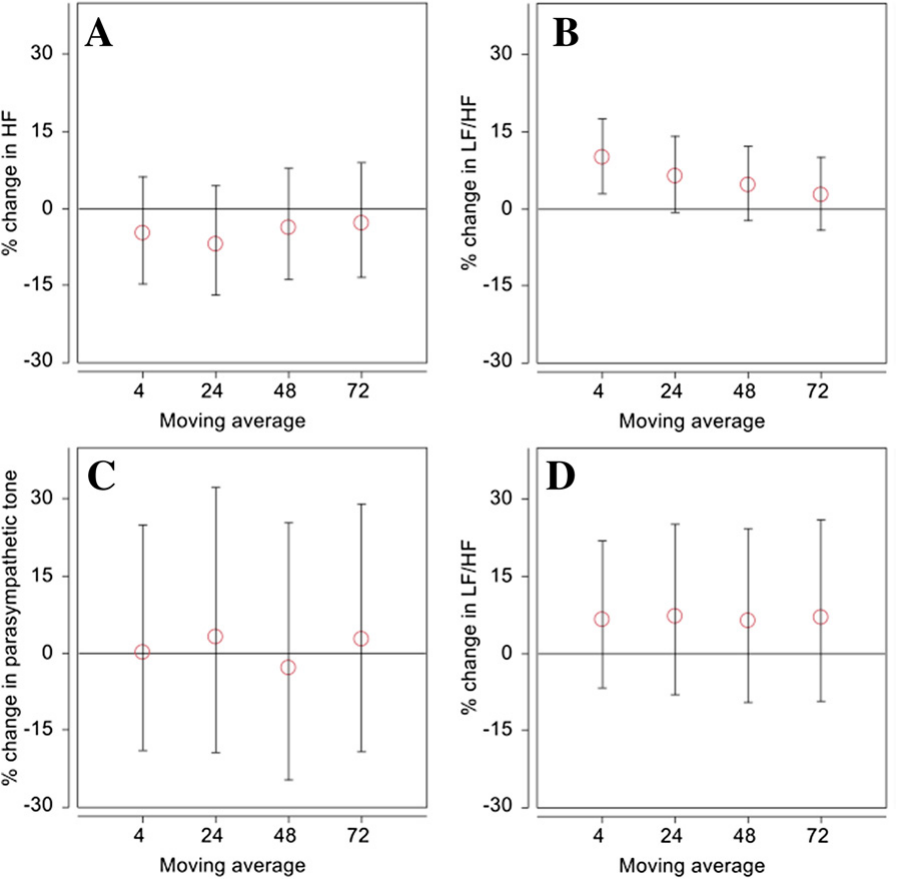
**Comparison of estimated percentage change in health effect outcome associated with an interquartile range (IQR) increase in either black carbon (BC) or traffic exposure at different moving averages (MA). (A)** BLMM: effect of BC on high frequency HRV (HF), **(B)** BLMM: effect of BC on sympathetic tone marker, low frequency to high frequency ratio (LF/HF), **(C)** SEM: effect of traffic on parasympathetic tone, and **(D)** SEM: effect of traffic on LF/HF. Error bars indicate 95% posterior interval.

Ordinary LMMs were fitted to double-check the estimates and posterior intervals from the BLMMs. The results from BLMMs and LMMs produced almost identical estimates and 95% intervals (results not shown here). Figure 4 compares the posterior mean and 95% PI from SEMs to the mean and 95% PI from BLMMs. For Parasympatheric tone, results generally showed larger effect estimates (more negative posterior means) for BLMMs as compared to SEMs. For example in a 4-hr pre-visit exposure, an IQR increase in BC was associated with changes of −4.8% (95% PI: -14.8 to 6.3%) in HF for BLMM as compare to a 0.1% change (95% PI: -19.0 to 25.0%) in Parasympathetic tone for an IQR increase in BC (reference pollutant for traffic pollution) for SEM. For sympathetic tone, comparison of the SEMs and BLMMs results showed that the effect estimates (posterior means) were all positive for both models. In contrast to that observed for the SEMs, BLMM showed a stronger association between 4-hr BC and LF/HF, with an increase of 10.1% (95% PI: 3.0 to 17.6%) in LF/HF per IQR increase in BC. Further, estimated variances of the SEMs were larger compared to the variances of the BLMMs, as evidenced by the wider estimated posterior intervals for SEMs as compared to BLMMs (see Table 3 for details).

## Discussion

In using SEMs, we found traffic pollution, as measured by BC, CO, NO, and NO_2_, to have null association with parasympathetic tone, as measured by HF, SDNN, and rMSSD, and weak positive association with sympathetic tone marker, LF/HF, at all the exposure windows. Our null association of traffic and parasympathetic tone, and the weak positive association of traffic and LF/HF are in agreement with the previous study of Park and coworkers that found no significant association between HRV markers and traffic-related pollutants, BC, NO_2_, and CO for any of the exposure averaging periods [11]. In addition, the previous study of Weichenthal and et al. did not also observe significant associations between BC and HRV [15] and from a controlled crossover study of diesel exhaust exposure [16]. However, our results are contrary to those from previous studies that showed associations between markers of traffic-related pollutants and HRV markers [4,17-22]. For example, Schwartz and coworkers found that exposure to traffic-related pollutant (e.g. BC) was associated with decreased SDNN and rMMSD response and associated with increased LF/HF response [4]. In addition, impacts assessed using the Bayesian linear mixed models (BLMMs) were associated with decreased parasympathetic tone marker, HF, and associated with increased sympathetic tone marker, LF/HF, and were consistent across all examined exposure windows, as evidenced by their respective estimated means (Table 3). Moreover, in the linear mixed modeling approach, we found a strong positive relationship between increased LF/HF and exposure to BC at the 4-hr (Posterior probability that *ρ*_*1*_ > 0 = 0.99) and 24-hr (Posterior probability *ρ*_*1*_ > 0 = 0.96) exposure windows, which was in agreement with the Schwartz et al. study that also showed a strong and positive association between BC and LF/HF [4].

The differences in the result we observed between the SEM approach and the Bayesian linear mixed model approach implies that the effect of traffic on parasympathetic is not the same as the effect of BC on HF (Table 3). The dissimilarity in results may be explained by the difference between traffic in general, which includes, CO, NOx from gas combustion vehicles, with modest BC emitted, and diesel particulate matter that is most closely associated with the BC variable. In 2010, Spira-Cohen and co-workers published a study that looked at the analyses of urban elemental carbon samples, although the study was not in Boston, the study found that over 90% was due to diesel emissions in particular [23]. The results from the Bayesian linear mixed model approach and the SEM approach would also imply the higher toxicity of diesel traffic emissions versus gasoline powered vehicle emissions. Our results showed that the examination of parasympathetic effects of traffic pollution is not needed for all the participants. As for the sympathetic tone outcome, in the SEM approach, traffic pollution was associated with increased LF/HF at all exposure windows. Moreover, in the linear mixed model approach, BC was also associated with increased LF/HF with the 4-hr and 24-hr exposure windows being strongly associated (Posterior probability that *ρ*_*1*_ > 0 is greater than 0.95).

Several works in the past have used factor analysis, a key component and the measurement model part of structural equation models, to relate multiple exposure variables with health variables. For example, the work of the EPA Workshop on Source Apportionment of Particulate Matter Health Effects summarized the results of time series analyses of health effects with source factors [24]. However, in our approach, we used both the measurement model part and the structural model part of SEMs. We created latent variables for traffic (exposure) and parasympathetic tone (outcome) and modeled the association dependencies between these two latent constructs.

Correspondingly, our observation of effect modification by diabetic status in the association between traffic pollution and parasympathetic tone may be due to a higher likelihood of diabetics to have decreased parasympathetic tone than non-diabetics [25] and/or to their greater baseline oxidative stress, which has been shown to mediate the autonomic effects of particles [26-28]. SEMs at all exposure windows showed a consistent pattern in the acute estimated effect of traffic on parasympathetic tone in diabetics. These results are supported by those from a population-based study that showed a high prevalence of decreased parasympathetic tone in type 2 diabetic participants [25]. Additionally, we observed evidence of effect modification by obesity on the association between traffic pollution exposure and decreased parasympathetic tone. Cardiac autonomic nervous system and obesity are related, Poirier et al. in 2006 reported that an increase in body weight is associated with a decline in parasympathetic tone and accompanied by a rise in mean heart rate [29]. This may explain why we found a stronger association between traffic pollution and parasympathetic tone among obese participants as compared to non-obese participants. In addition, when we stratified the participants to obese and non-obese, we still observed evidence of effect modification by diabetic status on the effect of traffic pollution on parasympathetic tone. However, no effect modification by obesity status was observed when we stratified the participants to diabetic and non-diabetic. Since only 8% of the participants were both obese and diabetic, it is unlikely in this case that obesity is acting as a surrogate for diabetes. Furthermore, we also observed evidence of effect modification by being both obese and diabetic versus non-obese and non-diabetic on the association between traffic pollution exposure and decreases parasympathetic tone (Table 4).

Our study to our knowledge is the first to demonstrate the simultaneous impacts of multiple traffic-related pollutants on parasympathetic tone and sympathetic tone marker LF/HF, cardiovascular health outcomes that have the capability to predict cardiac morbidity and sudden cardiac death. Our study showed that multiple markers of traffic pollution and health impacts could be analyzed concurrently using SEMs. Moreover, in using Bayesian methods whether SEMs or linear mixed models, the estimates are easily interpreted and are useful quantities, an advantage of our proposed approach. Our study in a Bayesian framework that utilized SEMs was able to integrate data and to account for variations in one probabilistic framework. Additionally, SEMs have the ability to analyze unbalanced longitudinal data or repeated measures, with multiple outcomes and/or exposure markers, and benefit from a reduction in measurement error bias. In addition, our study used a random subject intercept which means that the contrasts are a mixture of within and between subjects; for this reason, confounding factor bias could be small relative to that in a purely cross-sectional study design [30]. The comprehensive structural equation modeling approach, however, is not required for study designs that only have one exposure variable and one measured outcome variable, for which linear regression model and linear mixed model will still be the favored method of analysis for cross-sectional studies, and repeated and longitudinal studies, respectively. Nonetheless, more studies that look at the parallel use of the two approaches (linear mixed models and SEMs) are needed to verify and validate our results.

There are a number of limitations in our study. In our study, exposures were estimated using concentrations measured at several ambient monitoring sites for CO, NO and NO_2_ and at a single ambient monitoring site for BC, which will not capture spatial variation in air pollutant concentrations. Because participants lived up to 22 km on median straight line distance from our ambient monitoring site, this spatial variation may result in exposure measurement error that would probably be non-differential and could bias the results in either direction [31]. Additionally, bias due to unmeasured or residual confounding and predictor factors cannot be ruled out, and the potential for larger variance and wider posterior interval should also be considered if SEMs and not linear mixed models are to be used in air pollution epidemiologic studies [9,32]. Lastly, since the study population consists of a high percentage of older ever smoker males who are mostly veterans and predominately white that have particular occupational exposure; the results may not be generalizable to women, younger individuals, or other ethnic and racial groups. The effect of traffic pollution on parasympathetic tone and LF/HF on these other populations should be tackled in future studies.

## Conclusions

In conclusion, this study documented the null association between exposure to traffic pollution and parasympathetic tone and the weak association between traffic pollution exposure and sympathetic tone marker, LF/HF, health effect outcomes that have the capability to predict cardiovascular disease, among older men. In addition, BC is adversely associated with LF/HF in the elderly and that traffic pollution may decrease parasympathetic tone among diabetic elderly. The study also showed that SEMs in a Bayesian framework would be a plausible alternative method for unbalanced longitudinal and repeated measures studies in air pollution epidemiology that look at the cumulative effect of traffic pollution exposure on multiple health effect outcomes.

## Abbreviations

AT: Ambient temperature; ATemp: Apparent temperature; BC: Black carbon; BLMM: Bayesian linear mixed model; BMI: Body mass index; CO: Carbon monoxide; DAG: Directed acyclic graph; DM: Diabetes mellitus; DPT: Dew point temperature; HF: High frequency; HRV: Heart rate variability; IQR: Inter quartile range; LMM: Frequentist linear mixed model; LF: Low frequency; LF/HF: Low frequency/high frequency ratio; NAS: Normative Aging Study; NO2: Nitrogen dioxide; NO: Nitrogen monoxide; PI: Posterior interval; rMSSD: Square root of the mean squared differences of successive normal-to-normal intervals; SDNN: 5-minutes standard deviation of normal-to-normal intervals; SEM: Structural equation model.

## Competing interests

The authors declare that they have no competing interests.

## Authors’ contributions

ESB designed the study with support from HHS, JDS, BAC, GAW and PSV, conducted the analysis under the guidance of BAC, and wrote the manuscript with assistance from HHS. JDS, BAC, GAW, and PSV reviewed the drafts. All authors read and approved the final manuscript.

## Supplementary Material

Additional file 1 Implementation particulars and descriCSption of prior distributions of parameters. Click here for file

Additional file 2 WinBUGS Code. Click here for file

Additional file 3: Table S1 Adjusted posterior estimates of % change in mean sympathetic tone marker (LF/HF) associated with an IQR increase in traffic, by patient characteristics [diabetic status (doctor’s diagnosis of diabetes or fasting blood glucose (FBG) >126 mg/dL, vs. no diagnosis or FBG ≤126 mg/dL), and obesity (body mass index (BMI) ≥ 30, <30)]. Click here for file
