# Supplementary material for: Structural equation modeling of parasympathetic and sympathetic response to traffic air pollution in a repeated measures study
Source: Environ Health. 2013 Sep 23;12:81. doi: 10.1186/1476-069X-12-81 (PMC3907044; doi:10.1186/1476-069X-12-81)
Supplement: Additional file 1 — Implementation particulars and descriCSption of prior distributions of parameters. [file 1476-069X-12-81-S1.pdf]

ADDITIONAL FILE 1

Implementation Particulars

The variances of the measured exposure and outcome responses and latent exposure and outcome variables all had an inverse gamma prior distribution [*IG* (0.01, 0.01)]. In Equations (4), (9), (10), (11) and (12), regression coefficients all had a normal prior distribution [*N* (0, 1000)]. Moreover, all the subject level random intercepts, b_i, in Eq. (9 to 12) were considered to have normal priors with mean 0 and variance τ^b, where $\tau^b \sim IG\,(0.01, 0.01)$. Finally, given the fact that factor loadings/source contributions of traffic-related pollutants, and parasympathetic tone markers cannot be negative, the normal distribution on the factor loading parameters (α_l's and λ_l's) was truncated, $\alpha_l \sim N\,(0, 1000)\, I\,(\alpha_l \geq 0)$ and $\lambda_l \sim N\,(0, 1000)\, I\,(\lambda_l \geq 0)$.

Posterior distributions of parameters were obtained by using the Markov Chain Monte Carlo (MCMC) methods [1]. Using an interface with R (R2WinBUGS), an Open Source system for graphics and statistical computing (http://www.r-project.org), the methods were implemented in WinBUGS 1.4.3 software [2]. The MCMC algorithm was run for 2.15 million iterations using 3 chains; to assess burn-in, the first 2 million draws were used. Inferences were based on every 30th sample and 5000 samples from the posterior distribution were saved to generate approximately independent samples from the posterior distributions of the model parameters. Sample traces suggested convergence to the stationary distribution.

REFERENCES

1. Gelman A., Carlin J.B., Stern H.S., and Rubin D.B. Bayesian Data Analysis, 2nd edition. Chapman and Hall, New York, NY, 2003.

2. Spiegelhalter D., Thomas A., Best N., and Lunn D. WinBUGS Version 1.4.3. MRC Biostatistics Unit, Institute of Public Health: Cambridge, UK, 2006.
