# Supplementary material for: Structural equation modeling of parasympathetic and sympathetic response to traffic air pollution in a repeated measures study
Source: Environ Health. 2013 Sep 23;12:81. doi: 10.1186/1476-069X-12-81 (PMC3907044; doi:10.1186/1476-069X-12-81)
Supplement: Additional file 2 — WinBUGS Code. [file 1476-069X-12-81-S2.pdf]

ADDITIONAL FILE 2. WinBUGS Code

MODEL 1

```
############################################################
# Bayesian SEM: Effect of traffic on parasympathetic tone #
############################################################

model{

#########################################
# Data for parasympathetic tone outcome #
#########################################

  for(i in 1:N.resp){
    for(h in 1:N.out){
      outcome[i,h]<-NAS.responses[i,h]
    }
  }

#############################
# Data for traffic exposure #
#############################

  for(i in 1:N.resp){
    for(p in 1:P){
      exp[i,p]<-exposure[i,p]
    }
  }

################################
# Data for confounding factors #
################################

  for(i in 1:N.resp){
    for(c in 1:C){
      cov[i,c]<-covariates[i,c]
    }
  }

#######################
# Data for predictors #
#######################

  for(i in 1:N.resp){
    for(r in 1:R){
      pred[i,r]<-predictors[i,r]
    }
  }
```

```
#####################################################################
# Model for latent parasympathetic tone outcome regression         #
#####################################################################

  for(i in 1:N.resp){
    for(h in 1:N.out){
      outcome[i,h]~dnorm(mean.out[i,h],tau.out[h])
      mean.out[i,h]<-alpha0[h]+alpha1[h]*eta[i]
    }
    eta[i]~dnorm(mean.eta[i],tau.eta)
    mean.eta[i]<-gamma*zeta[i]+inprod(omega[1:C],cov[i,])+
                 inprod(nu[1:R],pred[i,])+b.idout[NAS.idd[i]]
  }

################################################
# Model for latent traffic exposure regression #
################################################

  for(i in 1:N.resp){
    for(p in 1:P){
      exp[i,p]~dnorm(mean.traf[i,p],tau.exp[p])
      mean.traf[i,p]<-lambda0[p]+lambda1[p]*zeta[i]
    }
    zeta[i]~dnorm(mean.zeta[i],tau.zeta)
    mean.zeta[i]<-inprod(psi[1:C],cov[i,])
  }

######################################################################
# priors in the eta (parasympathetic) and zeta (traffic) regression #
######################################################################

  gamma~dnorm(0, 0.001)

  for(c in 1:C){
    omega[c]~dnorm(0,0.001)
  }

  for(c in 1:C){
    psi[c]~dnorm(0,0.001)
  }

  for(r in 1:R){
    nu[r]~dnorm(0, 0.001)
  }

  for(d in 1:N.id){
    b.idout[d]~dnorm(0,tau.idout)
  }

  tau.idout~dgamma(0.01, 0.01)
  sigsq.idout<-1/tau.idout

  tau.eta~dgamma(0.01, 0.01)
  sigsq.eta<-1/tau.eta

  tau.zeta~dgamma(0.01, 0.01)
  sigsq.zeta<-1/tau.zeta
```

```
#######################################################
# Latent model for parasympathetic: alpha0 and alpha1 #
#######################################################

  for(h in 1:N.out){
    alpha0[h]~dnorm(0, 0.001)
    tau.out[h]~dgamma(0.01, 0.01)
    sigsq.out[h]<-1/tau.out[h]
  }

  alpha1[1]<-1

  for(h in 2:N.out){
    alpha1[h]<-alpha1.free[h-1]
    alpha1.free[h-1]~dnorm(0, 0.001) I(0,)
  }

#########################################################
# Latent model for traffic pollution: lambda0 + lambda1 #
#########################################################

  for(p in 1:P){
    lambda0[p]~dnorm(0, 0.001)
    tau.exp[p]~dgamma(0.01, 0.01)
    sigsq.exp[p]<-1/tau.exp[p]
  }

  lambda1[1]<-1

  for(p in 2:P){
    lambda1[p]<-lambda1.free[p-1]
    lambda1.free[p-1]~dnorm(0, 0.001) I(0,)
  }
}
```

MODEL 2

```
##################################################################
# Bayesian SEM: Effect of traffic on sympathetic tone marker LF/HF #
##################################################################

model{

###########################################
# Data for sympathetic tone outcome LF/HF #
###########################################

  for(i in 1:N.resp){
      outcome[i]<-NAS.responses[i]
  }

#############################
# Data for Traffic Exposure #
#############################

  for(i in 1:N.resp){
    for(p in 1:P){
      exp[i,p]<-exposure[i,p]
    }
  }

################################
# Data for confounding factors #
################################

  for(i in 1:N.resp){
    for(c in 1:C){
      cov[i,c]<-covariates[i,c]
    }
  }

#######################
# Data for predictors #
#######################

  for(i in 1:N.resp){
    for(r in 1:R){
      pred[i,r]<-predictors[i,r]
    }
  }

###################################################
# model for sympathetic tone outcome LF/HF regression #
###################################################

  for(i in 1:N.resp){
      outcome[i]~dnorm(mean.out[i],tau.out)
    mean.out[i]<-gamma*zeta[i]+inprod(omega[1:C],cov[i,])+
                 inprod(nu[1:R],pred[i,])+b.idout[NAS.idd[i]]
  }
```

```
################################################
# model for latent traffic exposure regression #
################################################

  for(i in 1:N.resp){
    for(p in 1:P){
      exp[i,p]~dnorm(mean.traf[i,p],tau.exp[p])
      mean.traf[i,p]<-lambda0[p]+lambda1[p]*zeta[i]
    }
    zeta[i]~dnorm(mean.zeta[i],tau.zeta)
    mean.zeta[i]<-inprod(psi[1:C],cov[i,])
  }

#######################################################################
# priors in the sympathetic tone marker and zeta (traffic) regression #
#######################################################################

  gamma~dnorm(0, 0.001)

  for(c in 1:C){
    omega[c]~dnorm(0,0.001)
  }

  for(c in 1:C){
    psi[c]~dnorm(0,0.001)
  }

  for(r in 1:R){
    nu[r]~dnorm(0, 0.001)
  }

  for(d in 1:N.id){
    b.idout[d]~dnorm(0,tau.idout)
  }

  tau.idout~dgamma(0.01, 0.01)
  sigsq.idout<-1/tau.idout

  tau.out~dgamma(0.01, 0.01)
  sigsq.out<-1/tau.out

  tau.zeta~dgamma(0.01, 0.01)
  sigsq.zeta<-1/tau.zeta

#########################################################
# latent model for traffic pollution: lambda0 + lambda1 #
#########################################################

  for(p in 1:P){
    lambda0[p]~dnorm(0, 0.001)
    tau.exp[p]~dgamma(0.01, 0.01)
    sigsq.exp[p]<-1/tau.exp[p]
  }

  lambda1[1]<-1
```

```
for(p in 2:P){
    lambda1[p]<-lambda1.free[p-1]
    lambda1.free[p-1]~dnorm(0, 0.001) I(0,)
  }
}
```

MODEL 3

```
####################################################################
# Bayesian LMM: Effect of traffic-related pollution on HRV marker #
####################################################################

model{

###############################
# Data for HRV marker outcome #
###############################

  for(i in 1:N.resp){
      outcome[i]<-NAS.responses[i]
  }

###############################################
# Data for traffic-related pollution exposure #
###############################################

  for(i in 1:N.resp){
      exp[i]<-exposure[i]
  }

################################
# Data for confounding factors #
################################

  for(i in 1:N.resp){
    for(c in 1:C){
      cov[i,c]<-covariates[i,c]
    }
  }

###########################################
# model for linear mixed model regression #
###########################################

  for(i in 1:N.resp){
      outcome[i]~dnorm(mean.out[i],tau.out)
      mean.out[i]<beta0+gamma*exp[i]+
                  inprod(omega[1:C],cov[i,])+b.idout[NAS.idd[i]]
  }

################################
# priors in the LMM regression #
################################

  gamma~dnorm(0, 0.001)

  beta0~dnorm(0, 0.001)

  for(c in 1:C){
    omega[c]~dnorm(0,0.001)
  }
```

```
  for(d in 1:N.id){
    b.idout[d]~dnorm(0,tau.idout)
  }

  tau.idout~dgamma(0.01, 0.01)
  sigsq.idout<-1/tau.idout

  tau.out~dgamma(0.01, 0.01)
  sigsq.out<-1/tau.out

}
```
