# Supplementary material for: Structural equation modeling of parasympathetic and sympathetic response to traffic air pollution in a repeated measures study
Source: Environ Health. 2013 Sep 23;12:81. doi: 10.1186/1476-069X-12-81 (PMC3907044; doi:10.1186/1476-069X-12-81)
Supplement: Additional file 3: Table S1 — Adjusted posterior estimates of % change in mean sympathetic tone marker (LF/HF) associated with an IQR increase in traffic, by patient characteristics [diabetic status (doctor’s diagnosis of diabetes or fasting blood glucose (FBG) >126 mg/dL, vs. no diagnosis or FBG ≤126 mg/dL), and obesity (body mass index (BMI) ≥ 30, <30)]. [file 1476-069X-12-81-S3.pdf]

Additional File 3

Additional Table 1. Adjusted posterior estimates of % change in mean sympathetic tone marker (LF/HF) associated with an IQR[a] increase in traffic, by patient characteristics [diabetic status (doctor's diagnosis of diabetes or fasting blood glucose (FBG) >126 mg/dL, vs. no diagnosis or FBG ≤126 mg/dL), and obesity (body mass index (BMI) ≥ 30, <30)].

STRUCTURAL EQUATION MODEL									
Effect Modifier	Moving average	% change in mean	95% PI	Posterior probability [b]	Effect Modifier	Moving average	% change in mean	95% PI	Posterior probability [b]
Diabetic	4-hr	-0.3	(-24.1, 30.8)	0.49	Diabetic	4-hr	-28.2	(-52.1, 6.8)	0.05
	24-hr	7.5	(-22.8, 49.4)	0.67	Obese	24-hr	-10.6	(-41.9, 39.9)	0.31
	48-hr	9.0	(-21.5, 50.7)	0.70		48-hr	1.8	(-33.2, 58.5)	0.53
	72-hr	16.8	(-16.1, 64.0)	0.82		72-hr	11.6	(-27.8, -76.8)	0.68
Non-Diabetic	4-hr	8.0	(-6.5, 24.3)	0.86	Non-Diabetic	4-hr	15.3	(-11.9, 51.0)	0.85
	24-hr	7.1	(-9.5, 26.3)	0.79	Obese	24-hr	4.9	(-23.4, 41.0)	0.62
	48-hr	5.8	(-10.8, 25.5)	0.75		48-hr	-1.5	(-30.5, 38.2)	0.47
	72-hr	5.2	(-11.1, 25.0)	0.72		72-hr	0.1	(-29.3, 43.0)	0.50
Obese	4-hr	0.0	(-20.7, 24.7)	0.50	Diabetic	4-hr	32.1	(-9.2, 91.3)	0.93
	24-hr	-0.1	(-22.8, 28.2)	0.50	Non-Obese	24-hr	32.5	(-16.9, 110.8)	0.88
	48-hr	0.1	(-23.9, 30.0)	0.51		48-hr	18.7	(-26.1, 93.6)	0.75
	72-hr	4.4	(-21.9, 39.3)	0.62		72-hr	22.3	(-25.1, 100.7)	0.79
Non-Obese	4-hr	9.4	(-5.7, 27.8)	0.88	Non-Diabetic	4-hr	5.7	(-10.8, 24.7)	0.74
	24-hr	10.1	(-8.1, 31.2)	0.86	Non-Obese	24-hr	7.6	(-10.6, 29.9)	0.78
	48-hr	8.8	(-8.9, 29.7)	0.83		48-hr	7.6	(-9.7, 28.9)	0.78
	72-hr	8.0	(-9.6, 29.6)	0.80		72-hr	6.3	(-12.1, 28.6)	0.74

[a] Interquartile range (IQR): 4-hr = 0.88; 24-hr = 0.52; 48-hr = 0.43; 72-hr = 0.36;
[b] Posterior probability that $\gamma_1 > 0$.
